# Rifampicin-Resistance Mutations in the *rpoB* Gene in *Bacillus velezensis* CC09 have Pleiotropic Effects

**DOI:** 10.3389/fmicb.2017.00178

**Published:** 2017-02-13

**Authors:** Xun-Chao Cai, Huan Xi, Li Liang, Jia-Dong Liu, Chang-Hong Liu, Ya-Rong Xue, Xiang-Yang Yu

**Affiliations:** ^1^State Key Laboratory of Pharmaceutical Biotechnology, School of Life Sciences, Nanjing UniversityNanjing, China; ^2^Institute of Food Safety and Inspection – Jiangsu Academy of Agricultural SciencesNanjing, China

**Keywords:** *Bacillus velezensis*, RNA polymerase, rifampicin resistance, mutation, iturin A

## Abstract

Rifampicin resistance (Rif^r^) mutations in the RNA polymerase β subunit (*rpoB*) gene exhibit pleiotropic phenotypes as a result of their effects on the transcription machinery in prokaryotes. However, the differences in the effects of the mutations on the physiology and metabolism of the bacteria remain unknown. In this study, we isolated seven Rif^r^ mutations in *rpoB*, including six single point mutations (H485Y, H485C, H485D, H485R, Q472R, and S490L) and one double point mutation (S490L/S617F) from vegetative cells of an endophytic strain, *Bacillus velezensis* CC09. Compared to the wild-type (WT) strain (CC09), the H485R and H485D mutants exhibited a higher degree of inhibition of *Aspergillus niger* spore germination, while the H485Y, S490L, Q472R, and S490L/S617F mutants exhibited a lower degree of inhibition due to their lower production of the antibiotic iturin A. These mutants all exhibited defective phenotypes in terms of pellicle formation, sporulation, and swarming motility. A hierarchical clustering analysis of the observed phenotypes indicated that the four mutations involving amino acid substitutions at H485 in RpoB belonged to the same cluster. In contrast, the S490L and Q472R mutations, as well as the WT strain, were in another cluster, indicating a functional connection between the mutations in *B. velezensis* and phenotypic changes. Our data suggest that Rif^r^ mutations cannot only be used to study transcriptional regulation mechanisms, but can also serve as a tool to increase the production of bioactive metabolites in *B. velezensis*.

## Introduction

DNA-dependent RNA polymerase (RNAP) is an enzyme that is essential to life. The core of the bacterial RNAP consists of five subunits (α_2_ββ′ω). The RNAP associates with the transcription initiation factor, σ, to form the RNAP holoenzyme (Ebright, 2000). The antibiotic rifampicin, which is used to treat multiple types of bacterial infections, exerts its effect by inhibiting RNAP. The crystal structure and genetic and biochemical data suggest that rifampicin binds to RNAP at a site adjacent to its active center, thereby physically blocking the formation of phosphodiester bonds in the RNA backbone. Rifampicin inhibits any RNA extension greater than two or three nucleotides (Campbell and Holt, 2001).

Resistance to rifampicin (Rif^r^) arises from mutations in the *rpoB* gene, which encodes the β subunit of RNAP. These mutations decrease the affinity of RNAP for rifampicin (Xu et al., 2005). In *Escherichia coli*, the majority of resistance mutations in the RpoB protein are located in three clusters. Cluster I comprises amino acids (aa) 507–533, cluster II comprises aa 563–572, and cluster III comprises aa 687 (Jin and Gross, 1988; Goldstein, 2014). Homologous mutations to those in *E. coli* have been observed in the RpoB protein of many other bacteria, such as *Mycobacterium tuberculosis*, *Bacillus subtilis*, *Neisseria meningitidis*, *Staphylococcus aureus*, and *Streptomyces coelicolor* (Alifano et al., 2015). The majority of the identified mutations are in the cluster I region of RpoB (Goldstein, 2014).

Rif^r^ mutations have pleiotropic phenotypes due to their effects on transcription (Jin et al., 1988a,b; Maughan et al., 2004). For instance, the Rif^r^ mutations S531F, Δ532A, L533P, and T563P in RpoB of *E. coli* lead to a hyper-temperature-sensitive phenotype in dnaA46 and rpoD800 backgrounds (Jin and Gross, 1991; Zhou and Jin, 1997). In addition, the Rif^r^ mutations Q469R, H482R, and S487L in *B. subtilis* have global effects on growth rate, competence for transformation, sporulation, and germination (Maughan et al., 2004; Perkins and Nicholson, 2008). Moreover, the Rif^r^ mutations S442L and H437R in *Streptomyces* spp. lead to a stringent response and an increase in antibiotic production (Hu et al., 2002). Therefore, Rif^r^ mutations have been used to better understand the regulatory mechanisms that are underlying bacterial physiology and virulence, and to manipulate gene expression (for strain improvement and drug discovery) in bacterial species that are of industrial interest (Alifano et al., 2015).

*Bacillus velezensis* is a relatively novel species. It was first described by Wang L.T. et al. (2008) as a heterotypic synonym of *Bacillus amyloliquefaciens*. Recently, based on comparative genomics and DNA relatedness calculations, *B. amyloliquefaciens* subsp. *plantarum*, *Bacillus methylotrophicus*, and *Bacillus oryzicola* were reclassified as heterotypic synonyms of *B. velezensis* (Dunlap et al., 2015). The distinctive characteristics of *B. velezensis* include methanol utilization, plant growth promotion, and biocontrol capacity (as a result of the production of multiple antibiotics) (Dunlap et al., 2015). Although, extensive research has been conducted on this species to increase its production of bioactive metabolites (Koumoutsi et al., 2007; Jha et al., 2016; Suthar and Nerurkar, 2016) and/or plant colonization capacity (Wang et al., 2014), it remains unclear whether its biocontrol capacity can be enhanced by mutating the global transcription machinery, RNAP, to produce beneficial biocontrol phenotypes.

In this study, we obtained seven spontaneous mutations in *rpoB* that confer resistance to rifampicin in an endophytic strain of *B. velezensis*, CC09. This strain produces the non-ribosomal peptide iturin A (a secondary metabolite with antibacterial and antifungal properties), and it exhibits broad antifungal activity against several phytopathogens, including *Glomerella glycines*, *Rhizoctonia solani*, and *Alternaria alternata* (Cai et al., 2013; Yang et al., 2014). We also evaluated the effects of these mutations on cell growth, pellicle formation, swarming motility, sporulation, and iturin A production.

## Materials and Methods

### Bacterial Strains and Media

The strain used in this study was *B. velezensis* CC09, which was previously isolated from an evergreen tree, *Cinnamomum camphora*, and deposited in the China General Microbiological Culture Collection Center (CGMCC no. 4669).

The medium used for *B. velezensis* cultivation was either Luria-Bertani (LB) medium (5 g/L yeast extract, 10 g/L tryptone, and 10 g/L NaCl; pH 7.0) or Gailiang LB (GLB) medium (3.75 g/L yeast extract, 11.25 g/L tryptone, 5 g/L starch, and 1 g/L NaCl; pH 7.0). The medium used for the fungi was potato-sucrose (PS) medium (200 g/L potato and 20 g/L sucrose). The solid media were prepared by adding 7–20 g/L agar to the liquid media.

### Screening of Rif^r^ Mutants and Mapping of the Mutations in the *rpoB* Gene

The wild-type (WT) strain was cultured overnight and spread on LB agar plates (200 μL/plate) containing 50 μg/mL rifampicin. The plates were incubated at 37°C for 48 h to generate spontaneous Rif^r^ mutants. The mutants were cultured in the LB medium containing rifampicin and then stored at -80°C.

Three pairs of primers (Supplementary Table S1) were used for PCR amplification of the *rpoB* gene. The samples were initially denatured at 94°C for 5 min, followed by 30 cycles of denaturation at 94°C for 30 s, annealing at 55°C for 30 s, extension at 72°C for 2 min, and a final extension at 72°C for 10 min. The PCR products were purified using Axygen AxyPrep Mag PCR Clean-Up Kits (Axygen Biosciences, Inc., Union City, CA, USA), sequenced by BGI (Shenzhen, China), and analyzed using BioEdit software (Hall, 1999) to identify the mutations in *rpoB*.

### Allelic Replacement Associated with the Point Mutations in *B. velezensis* CC09

To verify that the *rpoB* mutations identified in *B. velezensis* were sufficient to cause the Rif^r^ phenotypic change, the following experiments were performed. First, chromosomal DNA from six strains with single point mutation (H485Y, H485C, H485D, H485R, Q472R, S490L) and one strain with double point mutation (S490L/S617F) was prepared, and then it was amplified using PCR with the primer pair PMRif1/PMRif2 (Supplementary Table S2) flanking the mutated nucleotide, including upstream (∼800 bp) and downstream (∼1000 bp) sequences (Supplementary Figure S1).

For each mutation, the PCR product was ligated to a pMAD vector (a temperature-sensitive integrative plasmid) that had been digested by EcoR I/Bgl II using an In-Fusion HD Cloning Kit (Clontech Laboratories, Inc., Mountain View, CA, USA) (Sleight et al., 2010). The constructed plasmid, pMADrif, was introduced into competent cells of the WT strain by electroporation. Transformants were obtained after 12 h at 30°C on LB plates containing 5 μg/mL erythromycin. A pool of individual clones was cultured at 40°C in the LB medium containing the same concentration of erythromycin that used in the stationary phase. Two more cycles of growth were carried out by diluting the stationary phase culture into antibiotic-free fresh LB medium (Arnaud et al., 2004). Finally, single colonies were obtained by plating the diluted culture onto LB agar plates free of antibiotics. The bacteria were further screened using a replica plating technique with LB agar plates containing antibiotics (erythromycin or rifampicin) to obtain erythromycin-sensitive but rifampicin-resistant colonies. The colonies were confirmed by sequencing. Regarding the S617F mutation (phenylalanine instead of serine) at 1849 bp that was associated with the double mutant, the mutation was prepared using PCR with two primer pairs (PMRif1/PMRif3 and PMRif4/PMRif2) (Supplementary Table S2). The mutation was cloned into a pMAD vector that was subsequently introduced into competent WT cells.

### Determination of Minimum Inhibitory Concentration (MIC)

For each mutant, the minimum inhibitory concentration (MIC) of rifampicin was measured using a previously described method (Clinical and Laboratory Standards Institute [CLSI], 2012).

### Observation of Multicellular Growth Pattern

The WT strain and Rif^r^ mutants were cultured overnight. Subsequently, 1 mL of each culture was centrifuged at 14,940 × *g* for 10 min. For each strain, the pellet was resuspended in an appropriate amount of phosphate buffer (pH 7.0) to reach a concentration of approximately 1 × 10^8^ cells/mL. The resuspended cells were placed at the center of GLB plates (5 μL/plate) containing 2% agar. The plates were incubated at 32°C for 24 h. The multicellular growth pattern of each mutant was photographed.

### Measurements of Growth Rate, Swarm Motility, and Sporulation

The vegetative cells of the WT strain and Rif^r^ mutants were cultured in liquid GLB at a temperature of 32°C and a rotational speed of 120 rpm for 10 h for use in the following experiments.

#### Growth Rate

To assess the growth rate, 1% (vol/vol) of the culture was inoculated in 100 mL GLB at 32°C and 120 rpm in a 250-mL flask for 26 h. In order to plot a growth curve, 1 mL of the culture was taken every 30 min, and the optical density at 600 nm (OD_600_) was measured using a spectrophotometer (GENESYS 10S UV-Vis Spectrophotometer, Thermo Fisher Scientific, Inc., Madison, WI, USA). The specific growth rate (μ) was calculated using the following formula: $\mu =\frac{\text{2.303}\left(\text{lg }OD2-\text{lg }OD1\right)}{\left(t2-t1\right)}$

where μ represents the specific growth rate, and OD2 and OD1 represent the OD of the culture at sampling time t2 and t1, respectively.

#### Swarming Motility

To assess swarming motility, 5 μL of the culture was placed in the center of GLB plate containing 0.7% agar, followed by incubation at 32°C for 5 h. The swarming diameter was then measured. The mean swarming rate (mm/h) was calculated by dividing the swarming diameter (mm) by 5 (h) (Kearns and Losick, 2003).

#### Sporulation

To assess sporulation, the cell culture conditions that were used for the growth curve analysis were replicated. After 26 h, the numbers of spores inside and outside the cells (that were related to the vegetative cells) were counted using a hemocytometer (model XB-K-25, Shanghai Anxin Optical Instrument Manufacture, Co., Ltd, Shanghai, China) (Schaeffer et al., 1965). For each strain, three replicates were performed and a sporulation curve was plotted. The number of hours to 50% sporulation (S_50_) was calculated based on the sporulation curve.

### Determination of Inhibitory Activity against Fungal Spore Germination

For each strain, the ability of the culture filtrate to inhibit the germination of *Aspergillus niger* spores was assessed. Inhibition was calculated as the ratio of the number of colonies on the plates in the presence of the filtrate to the number of colonies in the absence of the filtrate. Culturing was carried out at 28°C for 2 days, based on a previously described method (Yang et al., 2014).

### Quantification of Iturin A

Approximately, 1% (vol/vol) of the overnight culture of each strain was inoculated in 50 mL GLB medium in a 250-mL flask at 32°C and 120 rpm for 48 h. Subsequently, 1 mL of the cell suspension was centrifuged at 14,940 × *g* for 10 min. The resultant supernatant was analyzed using high-performance liquid chromatography (HPLC), following a previously described method (Cai et al., 2013).

### Observation of Pellicle Formation

For each strain, the formation of pellicle was evaluated based on the method proposed by López and Kolter (2010). Briefly, overnight culture was centrifuged at 14,940 × *g* for 10 min. The pellet was resuspended in GLB to reach a concentration of 1 × 10^8^ cells/mL. The resuspended cells were added (1%, vol/vol) to 3 mL sterile GLB medium in the wells of a 12-well tissue culture plate. The cells were incubated at 32°C for 16 h to observe whether pellicle developed, and, if so, its appearance.

The pellicle weight was assessed in terms of its dry weight using a digital weighing balance (Sartorius TE1502S Talent Analytical Balance, Sartorius AG, Göttingen, Germany) according to a previously described method (Graba et al., 2013). Briefly, the pellicle was carefully harvested from each well using sterile pipette tips, rinsed gently with sterile distilled water, dried in an oven at 120°C until the weight stopped decreasing, and finally weighed using the digital balance.

### Quantitative Real-Time PCR (RT-qPCR) Analysis

Based on their antifungal activity, three strains were selected for RT-qPCR analysis: CC09, CC09-RIF5, and CC09-RIF3, that is, the WT strain, the S490L mutant (which exhibited decreased iturin A production), and the H485R mutant (which exhibited increased iturin A production), respectively. The strains were grown in GLB medium at 32°C until the pellicle was completely formed (16 h). Cells were harvested by centrifugation at 14,940 × *g* for 1 min and stored in liquid nitrogen. The total RNA was extracted using Invitrogen TRIzol Reagent (Thermo Fisher Scientific, Inc., USA). Reverse transcription was used to synthesize cDNA using a random primer from the TaKaRa RT-PCR Kit, D6110A (TaKaRa Bio, Inc., Kusatsu, Japan).

Sixteen *B. velezensis* CC09-specific genes were amplified: 15 that encode proteins associated with antibiotic production and pellicle formation, and the gene that encodes ribosomal 16S RNA (as the internal reference). The primers used for the amplification of the genes were designed according to the genome sequence of *B. velezensis* CC09 (GenBank no. CP015443) (Cai et al., 2016) (Supplementary Table S3).

For each mutant, the fold-change in the expression of each gene was calculated by dividing the value for the mutant by the value for the WT strain using a previously described method (Schmittgen and Livak, 2008). A fold-change >2- or <0.5 compared to the WT strain was considered to represent a significant degree of differential expression. The set of samples for each strain involved three biological and two technical replicates.

### Data Analysis

GraphPad Prism version 3.02 (GraphPad Software, Inc., San Diego, CA, USA, www.graphpad.com) software was used to assess the significance of the differences using one-way analysis of variance (ANOVA) and Tukey’s multiple comparison test (*p* < 0.05 was considered statistically significant). A dendrogram of hierarchical clustering was used to illustrate the possible correlation between the mutations in *rpoB* and the phenotypic changes using an R package for weighted correlation network analysis (Langfelder and Horvath, 2008).

## Results

### Spectrum of Rif^r^ Mutations in the *B. velezensis rpoB* Gene

A total of 14 spontaneous Rif^r^ mutants were isolated by plating the WT strain in plates containing rifampicin. Among these, we identified seven unique mutations in the *rpoB* gene. Four of the mutants (0901, 0926, 0928, and 0933) had mutations that were located at the same site in RpoB, at aa 485. These four mutations involved substitutions of histidine (H) for tyrosine (Y), cysteine (C), arginine (R), and aspartic acid (D), respectively. The mutation in the 0917 mutant was located at aa 490, and involved a substitution of serine (S) for leucine (L) (S490L), and the mutations in the 0943 mutant was located at aa 472, and involved a substitution of glutamate (Q) for arginine (R) (Q472R). The 0954 mutant had mutations at two sites in *rpoB*, one at aa 490 that involved a substitution of serine (S) for leucine (L) (S490L, as in one of the previous mutants), and the other at aa 617 that involved a substitution of serine (S) for phenylalanine (F) (S617F).

**FIGURE 1:**
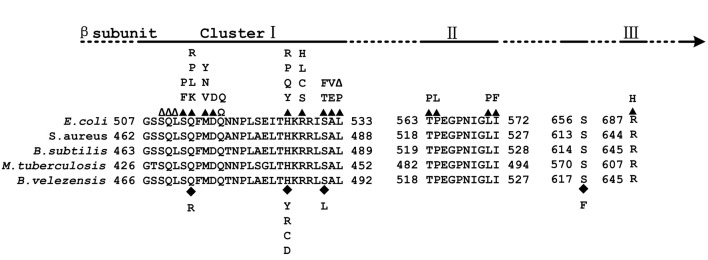
**Map of the *Escherichia coli* RNA polymerase β (RpoB) subunit. (Top)** Location of Rif clusters I, II, and III. **(Bottom)** Amino acid sequence alignment of the Rif clusters in *E. coli*, *Staphylococcus aureus*, *Bacillus subtilis*, *Mycobacterium tuberculosis*, and *Bacillus velezensis*. The numbering begins at the first amino acid of the RpoB sequences. Well-characterized RpoB substitutions that cause rifampicin resistance in *E. coli* are noted above the *E. coli* RpoB sequence. Closed triangles above the *E. coli* sequence indicate amino acid substitutions; empty triangles indicate amino acid deletions; Ω indicates amino acid insertions; closed diamonds below the *B. velezensis* sequence indicate amino acid substitutions.

To exclude the possibility that the observed phenotypic changes were related to secondary mutations rather than the mutations identified in *rpoB*, each of the mutations was cloned into a plasmid (pMAD) and introduced into the WT strain to generate a set of eight strains (seven with single mutations: H485Y, H485C, H485R, H485D, S490L, Q472R and S617F, and one with a double mutation: S490L/S617F) with mutations in *rpoB* but that were otherwise isogenic with the WT strain. Fortunately, all mutations, except S617F that was associated with the double mutant and insufficient to confer resistance to rifampicin, led to the Rif^r^ phenotype (**Table 1**). All the mutations that caused rifampicin resistance in vegetative *B. velezensis* CC09 cells were mapped to a rather restricted location within cluster I of RpoB (**Figure 1**).

**Table 1:** Point mutations in the *rpoB* gene of *Bacillus velezensis* that confer Rif^r^.

Strain	Nucleotide	Amino acid	Rif^r^	Number^∗^
CC09	NA	NA	-	NA
CC09-RIF1	C1453T	H485Y	+	2
CC09-RIF5	C1469T	S490L	+	2
CC09-RIF2	CA1453(4) TG	H485C	+	2
CC09-RIF3	A1454G	H485R	+	4
CC09-RIF4	C1453G	H485D	+	1
CC09-RIF6	A1415G	Q472R	+	2
CC09-RIF7	C1469T/C1850T	S490L/S617F	+	1
CC09-6I7F	C1850T	S617F	-	NA

*^∗^Number of spontaneous mutants containing the same point mutation in *rpoB* gene. CC09 is the WT strain of *B. velezensis*. + or – indicates the strain resistant or sensitive to Rif. NA means not applied in the experiment*.

Subsequently, seven mutants, labeled CC09-RIF1 (H485Y), CC09-RIF2 (H485C), CC09-RIF3 (H485R), CC09-RIF4 (H485D), CC09-RIF5 (S490L), CC09-RIF6 (Q472R), and CC09-RIF7 (S490L/S617F), were studied in detail in the following experiments.

### Effects of Rif^r^ Mutations on the Cell Growth, Physiology, and Metabolism of *B. velezensis* CC09

#### Multicellular Growth Pattern

**FIGURE 2:**
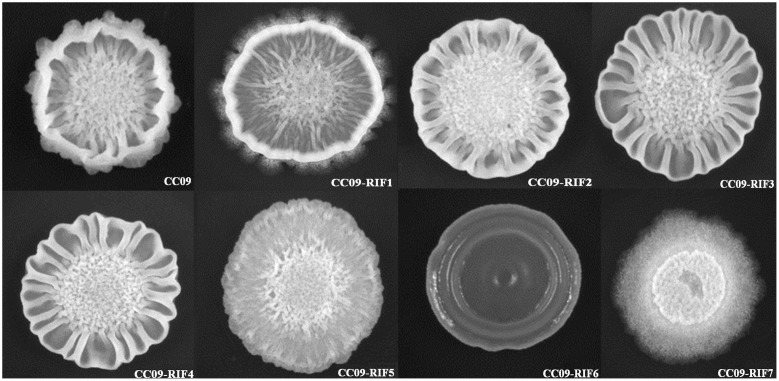
**Morphology of the multicellular growth patterns of the WT strain and Rif^r^ mutants**.

Compared to the WT strain, all seven Rif^r^ mutants exhibited a different multicellular growth pattern (**Figure 2**). The CC09-RIF2 (H485C), CC09-RIF3 (H485R), and CC09-RIF4 (H485D) strains had a mutation of the same histidine residue (H485) and exhibited similar multicellular growth patterns. However, the point mutation of the same residue from histidine (H) to tyrosine (Y) (CC09-RIF1) caused an intermediate phenotype (which was like the phenotypes of both the WT strain and the other mutants containing H485 mutations). CC09-RIF6 (Q472R) exhibited a very smooth multicellular growth pattern, while CC09-RIF7 (S490L/S617F) exhibited a “powdery” multicellular growth pattern in the same culture conditions. These results clearly indicate that the mutations in the *rpoB* gene that confer resistance to rifampicin dramatically altered the multicellular growth pattern of *B. velezensis.*

#### Growth Rate

OD_600_ values were used to determine the specific growth rate of the strains (**Table 2**). A slow specific growth rate in comparison to the WT strain (*p* < 0.05) was exhibited by CC09-RIF7 (S490L/S617F). In contrast, the other Rif^r^ mutants exhibited the same specific growth rate as the WT strain (*p* > 0.05). In other words, in the given culture conditions, the growth rate of all the mutants with single point mutations in *rpoB* (i.e., H485Y, H485C, H485D, H485R, S490L, Q472R, and S490L) were not significantly affected. However, compared to the effect of S490L in CC09-RIF5, the second mutation, S617F, in the double mutant CC09-RIF7 (S490L/S617F) reduced the specific growth rate (*p* < 0.05). The mechanism by which the single point mutation, S617F, influences the phenotypes of *B. velezensis* and the mechanism by which S490L and S617F interact will be explored in a future study.

**Table 2:** Effects of the point mutations in the *rpoB* gene that confer Rif^r^.

Strain	Mutation	μ (OD/h)	SM (mm/h)	S_50_ (h)	SGI (%)	Iturin A (μg/ml)	MIC (μg/mL)
CC09	-	0.94 ± 0.04a	15.5 ± 0.6a	34.2	71.8 ± 1.4c	168.1 ± 15.2c	0
CC09-RIF1	H485Y	0.86 ± 0.06a	7.6 ± 0.3c	58.8	52.5 ± 2.2 b	136.7 ± 9.5d	1024
CC09-RIF2	H485C	1.04 ± 0.09a	10.4 ± 0.5b	48.6	72.4 ± 2.2c	184.9 ± 6.7c	1024
CC09-RIF3	H485R	0.90 ± 0.03a	9.6 ± 0.7b	52.4	80.9 ± 1.2d	342.1 ± 10.1a	1024
CC09-RIF4	H485D	0.95 ± 0.06a	9.5 ± 0.6b	55.9	83.4 ± 2.6d	280.9 ± 3.6b	1024
CC09-RIF5	S490L	0.91 ± 0.04a	2.9 ± 0.2d	78.7	40.4 ± 1.1a	73.7 ± 5.8e	128
CC09-RIF6	Q472R	0.90 ± 0.06a	9.3 ± 0.4b	84.1	48.6 ± 2.3b	93.8 ± 5.2e	256
CC09-RIF7	S490L/S617F	0.66 ± 0.01b	2.7 ± 0.1d	50.3	44.1 ± 2.4a	134.9 ± 4.0d	128

*Data in the table was presented as average value ± standard deviation (SD) of the values in three biological replicates, except the values of S_*50*_ and MIC; μ-specific growth rate; SM-swarming motility; S_*50*_-the time of 50% vegetative cells formed spores which were calculated according to the sporulation curve in Supplementary Figure S2; SGI-spore germination inhibition of *Aspergillus niger*; iturin A- Iturin A concentration in the culture; MIC-the minimum inhibitory concentration against the antibiotic rifampicin. Different lowercase letters on each number define the test strains (triplicate sets) that showed significant differences (*p* < 0.05) in one-way ANOVA and Tukey’s Multiple Comparison Test*.

#### Swarming Motility

In terms of swarming motility, compared to WT strain (15.5 mm/h), the Rif^r^ mutants all exhibited weak swarming motility when they were cultured on 0.7% GLB agar plates at 32°C for 5 h (**Table 2**). CC09-RIF5 (S490L) (2.9 mm/h) and CC09-RIF7 (S490L/S617F) (2.7 mm/h) exhibited the slowest swarming motility, followed by CC09-RIF1 (H485Y) (7.6 mm/h), and then CC09-RIF2 (H485C) (10.4 mm/h), CC09-RIF3 (H485R) (9.6 mm/h), CC09-RIF4 (H485D) (9.5 mm/h), and CC09-RIF6 (Q472R) (9.3 mm/h).

#### Sporulation

The effect of the Rif^r^ mutations on spore formation in *B. velezensis* was complicated by the potential effects of both the mutation site and the substituted aa. In general, the mutations all greatly delayed sporulation compared to that of the WT strain (Supplementary Figure S2). Based on the S_50_, the mutants could be roughly grouped into four classes. Class I contained two mutants [CC09-RIF5 (S490L) and CC09-RIF6 (Q472R)] with S_50_ of 78.7–84.1 h; class II contained one mutant [CC09-RIF1 (H485Y)] with an S_50_ of 58.8 h; class III contained two mutants [CC09-RIF3 (H485R) and CC09-RIF4 (H485D)] with S_50_ of 52.4–55.9 h; and class IV contained two mutants [CC09-RIF2 (H485C) and CC09-RIF7 (S490L/S617F)] with S_50_ of 48.6–50.3 h. In contrast, the S_50_ of the WT strain was 34.2 h. These data suggest that the Rif^r^ mutations in *rpoB* have severe impacts on the sporulation of *B. velezensis*, and potentially on the sporulation of other *Bacillus* species.

#### Pellicle Formation

Based on unaided observations at 7 h after inoculation, we found pellicle in the wells containing the WT strain and the CC09-RIF3 (H485R), CC09-RIF4 (H485D), CC09-RIF2 (H485C), and CC09-RIF1 (H485Y) mutants. However, the formation of pellicle in the wells containing the CC09-RIF7 (S490L/S617F), CC09-RIF6 (Q472R), and CC09-RIF5 (S490L) mutants was significantly delayed to 11, 13, and 15 h post-incubation, respectively.

**FIGURE 3:**
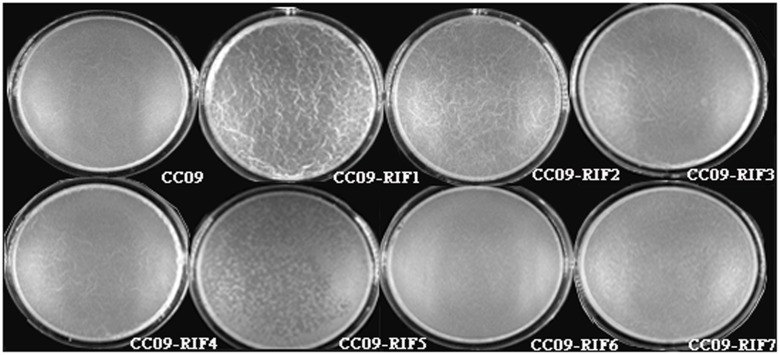
**Appearance of the pellicle of the WT strain and Rif^r^ mutants at 16 h after inoculation**.

Moreover, the appearance of the pellicle varied among the Rif^r^ mutants. For instance, the surface of the pellicle of the CC09-RIF3 (H485R), CC09-RIF4 (H485D), CC09-RIF2 (H485C), and CC09-RIF1 (H485Y) mutants was quite wrinkled and thick, while that of the CC09-RIF5 (S490L) and CC09-RIF7 (S490L/S617F) mutants, as well as the WT strain, was very smooth and thin (**Figure 3**). In addition, the weight of the pellicle produced by the CC09-RIF2 (H485C) and CC09-RIF4 (H485D) mutants was 6.0 mg and 2.8 mg, respectively, which was significantly different from that of the WT strain (4.5 mg) (Supplementary Figure S3). Due to the lower yield of pellicle, it was difficult to evaluate the weight of the pellicle produced by the CC09-RIF5 (S490L) and CC09-RIF6 (Q472R) mutants. These results indicate that Rif^r^ mutations in the *rpoB* gene have important effects on pellicle formation in *B*. *velezensis*.

#### Antifungal Activity

Based on the plate assay, the culture filtrate of the seven Rif^r^ mutants exhibited differences in their inhibition of *A. niger* spore germination (**Table 2**). The CC09-RIF3 (H485R) and CC09-RIF4 (H485D) mutants exhibited the highest antifungal activity (they inhibited 80.87–83.43% of spore germination); followed by CC09-RIF2 (H485C) (72.41%); CC09-RIF1 (H485Y) and CC09-RIF6 (Q472R) (48.58–52.49%); and CC09-RIF5 (S490L) and CC09-RIF7 (S490L/S617F) (40.36–44.09%). All the mutants except for CC09-RIF2 (H485C) exhibited a significant change (higher or lower) in their ability to inhibit spore germination compared to the WT strain (*p* < 0.05) (**Table 2**).

#### Iturin A Production

The different inhibitory effects of the culture filtrate on fungal spore germination are likely to be related to the level of iturin A production. Indeed, the production of iturin A by Rif^r^ mutants was positively correlated (*R*^2^ = 0.76, *p* < 0.01) with their antifungal activity (**Table 2**; Supplementary Figure S4). The CC09-RIF3 (H485R) and CC09-RIF4 (H485D) mutants produced a much larger quantity of iturin A compared to the WT strain, while the CC09-RIF1 (H485Y), CC09-RIF6 (Q472R), CC09-RIF7 (S490L/S617F), and CC09-RIF5 (S490L) mutants produced a significantly lower quantity of iturin A. The CC09-RIF2 (H485C) mutant was not significantly different compared to the WT strain in terms of iturin A production. Moreover, different aa substitutions at the same site in RpoB also significantly affected the production of iturin A. For instance, the concentration of iturin A in the cultures of the CC09-RIF1 (H485Y), CC09-RIF2 (H485C), CC09-RIF4 (H485D), and CC09-RIF3 (H485R) mutants (which had mutations at 485 that caused a substitution of H for Y, C, D, and R, respectively) were 136.7, 184.9, 280.9, and 342.1 μg/mL, respectively.

Rif^r^ mutations at different aa positions can have the same effect on iturin A production. For example, the production of iturin A did not differ significantly between CC09-RIF1 (H485Y) and CC09-RIF7 (S490L/S617F), or between CC09-RIF5 (S490L) and CC09-RIF6 (Q472R) (*p* > 0.05). However, the former pair exhibited a significantly higher production compared to the latter (*p* < 0.05). Moreover, by comparing the iturin A produced by the CC09-RIF5 (S490L) and CC09-RIF7 (S490L/S617F) mutants, we found that the S617F mutation had a significant compensatory effect on the reduced production of iturin A caused by the S490L mutation. These results suggest that mutations at different sites in the *rpoB* alleles or different aa substitutions corresponding to the same sites in the *rpoB* alleles influence iturin A production, which may be due to different mechanisms.

#### MIC of Rifampicin

The four mutants with different aa substitutions at position 485 in RpoB [CC09-RIF1 (H485Y), CC09-RIF2 (H485C), CC09-RIF3 (H485R), and CC09-RIF4 (H485D)] had the highest degree of rifampicin resistance among the seven Rif^r^ mutants, with a MIC of 1.024 mg/mL. In contrast, the mutation in the CC09-RIF5 (S490L) mutant and the mutation in the CC09-RIF6 (Q472R) mutant led to relatively weak degrees of rifampicin resistance, with a MIC of 0.128 and 0.256 mg/mL, respectively (**Table 2**).

#### Dendrogram of Hierarchical Clustering Based on the Phenotypes

**FIGURE 4:**
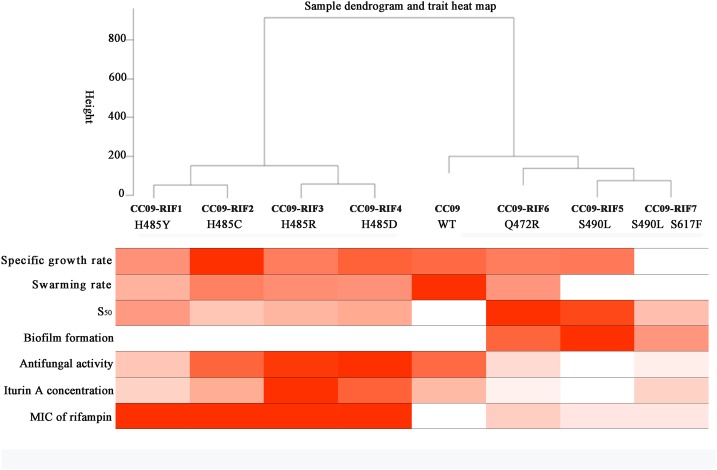
**Dendrogram of hierarchical clustering based on the phenotypes of the test strains**.

A clustering analysis of the data in **Table 2** was carried out to study the correlation between the Rif^r^ mutations and their corresponding phenotypes in *B. velezensis* CC09. When the cutline height was set to 250, the mutants containing aa substitutions at H485 were clustered into the same group, while the strains with S490L and Q472R mutations, as well as the WT strain, were clustered into another group (**Figure 4**). This result indicates that H485 is a key aa in the RpoB of *B. velezensis*, and any substitutions of this aa lead to a distinctive change in morphology, physiology, and metabolism. Compared to the H485 mutations, the S490L and Q472R mutations had relatively weak impacts on the biological processes of *B. velezensis*, as the strains with these mutations were clustered into the same group as the WT strain.

When the cutline height was set to 150, the test strains were clustered into five groups. Group 1 contained CC09-RIF1 (H485Y) and CC09-RIF2 (H485C), group 2 contained CC09-RIF3 (H485R) and CC09-RIF4 (H485D), group 3 contained CC09-RIF5 (S490L) and CC09-RIF7 (S490L/S617F), group 4 contained CC09-RIF6 (Q472R), and group 5 contained the WT strain (**Figure 4**).

These results strongly suggest that the mutations in the *rpoB* gene that cause resistance to rifampicin play important roles in the regulation of the biological processes in *B. velezensis.*

### Effects of Rif^r^ Mutations on Gene Expression

As the Rif^r^ mutations in the *rpoB* gene (which encodes the β subunit of RNAP) resulted in pleiotropic phenotypes in *B. velezensis*, 15 genes that encode proteins associated with antibiotic production and pellicle formation (Supplementary Table S3) were selected for RT-qPCR analysis to identify the potential correlation between the expression of the 15 genes and the phenotypic changes. We carried out RT-qPCR using three strains [i.e., WT, CC09-RIF3 (H485R), and CC09-RIF5 (S490L)]. Compared to the WT strain, the expression of four genes (i.e., *abrB*, *pgdS*, *cheV*, and *sinI*) was significantly up regulated in both mutants [CC09-RIF3 (H485R) and CC09-RIF5 (S490L)]. Three genes (i.e., *kinC*, *kinE*, and *yczE*) were up regulated in CC09-RIF3 (H485R) but unchanged in CC09-RIF5 (S490L), while two genes (i.e., *degS* and *degU*) were downregulated in CC09-RIF5 (S490L) but unchanged in CC09-RIF3 (H485R). These results indicate that the mutations in RpoB had wide-ranging effects on the transcription of the 15 functional genes (**Table 3**).

**Table 3:** Relative expression of 15 selected genes in the Rif^r^ mutants CC09-RIF5 (S490L) and CC09-RIF3 (H485R).

Gene	CC09-RIF5 (S490L)	CC09-RIF3 (H485R)
*degS*	0.32 ± 0.09	1.25 ± 0.47
*degU*	0.46 ± 0.04	1.62 ± 0.62
*abrB*	4.01 ± 0.82	2.02 ± 0.35
*pgdS*	3.36 ± 0.51	2.68 ± 0.33
*cheV*	8.23 ± 1.02	4.27 ± 0.04
*kinC*	1.88 ± 0.23	2.01 ± 0.10
*kinE*	0.72 ± 0.04	2.78 ± 0.22
*yczE*	0.63 ± 0.12	2.66 ± 0.54
*ituA*	0.67 ± 0.02	1.22 ± 0.12
*ituB*	0.82 ± 0.09	0.14 ± 0.01
*srfAB*	0.43 ± 0.03	0.24 ± 0.04
*srfAA*	1.48 ± 0.35	0.17 ± 0.08
*sinI*	3.23 ± 0.08	2.03 ± 0.25
*sinR*	1.56 ± 0.36	0.78 ± 0.02
*spo0A*	0.66 ± l.20	0.91 ± l.20

*Data in the table was presented as average value ± standard deviation (SD) of the value of three biological replicates*.

Moreover, based on the hierarchical clustering analysis of the relative expression of the 15 genes that were quantified using RT-qPCR, when the cutline height was set to 140, we found that CC09-RIF3 (H485R) belonged to an independent group, while CC09-RIF5 (S490L) and the WT strain were clustered into a separate group (Supplementary Figure S5). This is consistent with the hierarchical clustering results based on the phenotypes (**Figure 2**). These results demonstrate that the pleiotropic phenotypes of the Rif^r^ mutants were likely to be caused by changes in the global transcriptional regulation of the genes that exhibited differential expression.

## Discussion

The aim of this study was to isolate, sequence, and phenotypically characterize several Rif^r^ mutations in the *rpoB* gene of *B. velezensis*, an endophytic bacterium with biocontrol properties. We obtained six strains with single and one with a double point mutation in *rpoB* that conferred resistance to rifampicin (all of which were in the cluster I region of RpoB). These Rif^r^ mutations have been described in other bacteria, such as *E. coli* (Jin et al., 1988b), *S. lividans* (Hu et al., 2002), *B. subtilis* (Ingham and Furneaux, 2000), *M. tuberculosis* (Mokrousov et al., 2003), and *S. aureus* (Wichelhaus et al., 1999). Moreover, these Rif^r^ mutations in *B. velezensis* influence a wide range of processes, including cell growth, pellicle formation, swarming motility, sporulation, and iturin A production, which might be a result of the regulation of related genes.

Most of the Rif^r^ mutations that have been identified thus far map directly to the domain of, or regions adjacent to, the rifampicin-binding pocket of the RpoB (Jin and Gross, 1988, 1991; O’Neill et al., 2000). The aa substitutions at D516, H526, and S531 (using the *E. coli* RpoB numbering system) frequently confer high levels of resistance to refamipicin in Rif^r^ mutant clinical isolates (Alifano et al., 2015). Among the 14 Rif^r^ mutants sequenced in this study, eight (57.1%) had aa substitutions at H485, and two (14.3%) had aa substitutions at S490, which correspond to homologous substitutions at H526 and S531, respectively, in *E. coli* RpoB (**Table 1**). These results suggest that the frequency of Rif^r^ mutations in the *rpoB* gene of *B. velezensis* was probably similar to that in other bacteria such as *E. coli*. In addition, all the aa substitutions at H485 in RpoB of *B. velezensis* led to the highest MIC of rifampicin among the test strains (**Table 2**), which is consistent with the findings of a study by Alifano et al. (2015).

The substitution of the glutamine (Q), histidine (H), and serine (S) residues in the cluster I region of RpoB (Q513, H526, and S531 in *E. coli*; Q469, H482, and S487 in *B. subtilis*; Q472, H485, and S490 in *B. velezensis*) are the most effective mutations in terms of the promotion adaptive responses. They cause global changes in the bacteria, such as alternative substrate utilization, secondary metabolism, sporulation/germination, and antibiotic heteroresistance (Zhou and Jin, 1998; Maughan et al., 2004; Alifano et al., 2015). However, the effects of Rif^r^ mutations on phenotypes vary both within and between bacterial species. For instance, compared to the WT strain, the S487L mutation in the *rpoB* gene of *B. subtilis* led to a lower specific growth rate, while Q469R and H482R/Y mutations did not affect the growth of the strain when it was cultured in LB medium (Maughan et al., 2004). The Q469R and S487L mutations dramatically inhibited sporulation while H482R/Y exhibited a weak impact or no impact on spore formation in *B. subtilis* (Moeller et al., 2012). In this study, almost all of these mutations (Q469R, S487L, and H482R/Y) in *B. velezensis* exhibited inhibitory effects on spore formation.

Interestingly, we isolated a *B. velezensis* CC09 strain with a double point mutation (CC09-RIF7), which had S490L and S617F substitutions in RpoB. Although the S617F mutation found in the double mutant CC09-RIF7 was not sufficient to confer resistance to rifampicin, it exhibited compensatory effects on the phenotype defects (e.g., defects in pellicle formation, sporulation, and iturin A production) caused by the S490L mutation. This was probably due to a functional or structural interaction between the two sites (Jin and Gross, 1988).

According to the classification system proposed by Alifano et al. (2015), the Rif^r^ mutations in the cluster I region of RpoB of *B. velezensis* can be clustered into three groups: (i) H485 mutations that lead to a decrease in transcription termination; (ii) Q472 mutations that result in an increase in transcription termination; and (iii) S490 mutations that cause a decrease in antitermination. This classification is relatively consistent with the dendrograms of hierarchical clustering based on the phenotypes (**Figure 4**) and the relative expression of the 15 functional genes (Supplementary Figure S5) that we observed. Mutants with substitutions in RpoB at aa position H485 (H485Y, H485C, H485R, and H485D) were classified into a group with a high level of iturin A production and increased inhibition of fungal spore germination. In fact, several studies on multiple species revealed that H485 mutations are closely associated with the initiation of, and increase in, antibiotic production (Ingham and Furneaux, 2000; Hu et al., 2002; Wang G. et al., 2008; Tanaka et al., 2013). We intend to explore the correlations between the phenotypes (e.g., pellicle formation, sporulation, and iturin A production) and gene expression in a future study.

The effect of the Rif^r^ mutations on antibiotic production is dependent on the type of antibiotic and bacterial species. For example, the Rif^r^ mutations H437R and S442L significantly enhance the production of streptomycin by *S. griseus* (Tanaka et al., 2013), while homologous mutations inhibit the production of iturin A in *B. velezensis*. The H439R/D mutations in RpoB increase the production of erythromycin, while Q426R reduces the production of erythromycin in *Saccharopolyspora erythraea* (Carata et al., 2009). These mutations (i.e., H485R/D and Q472R) had similar effects in *B. velezensis* CC09 with respect to iturin A production (**Table 2**).

In order to understand the possible relationship between iturin A production and gene expression, we performed an RT-qPCR analysis of two genes, *ituA* and *ituB* in the iturin A operon, which encode synthetases in CC09-RIF3 (H485R), CC09-RIF5 (S490L), and WT strains of *B. velezensis.* Surprisingly, the production of iturin A (**Table 2**) was inconsistent with the expression of *ituA* and *ituB* in the mutants (**Table 3**). However, the relative expression of *yczE*, which encodes an integral membrane protein, in the CC09-RIF3 (H485R) strain was significantly higher than that in the CC09-RIF5 (S490L) and WT strains. YczE has been reported to be a candidate for anchoring the synthetases at the membrane and enhancing the production of bacillomycin D, which belongs to the iturin A family (Koumoutsi et al., 2007). Thus, the production of iturin A by *B. velezensis* may be regulated at the post-transcriptional stage (Koumoutsi et al., 2007).

Mature pellicles are embedded in an extracellular polymeric matrix, which is composed of exopolysaccharides, proteins, adhesins, and, occasionally, DNA (Luary et al., 2014). They have a significant impact in medical, industrial, and agricultural settings; for example, they can enhance bacterial colonization of plants (Hallstoodley et al., 2004). Many extracellular metabolites such as surfactants (e.g., iturins and surfactins), polysaccharides, γ-polyglutamic acid (PGA), and proteins (e.g., CheV) are associated with pellicle formation.

The formation of pellicles is mainly regulated via Spo0A/AbrB, SinI/SinR, and DegS/DegU regulatory systems in *Bacillus* species (Chu et al., 2008; Wang et al., 2015). Our RT-qPCR data (based on the analysis of 15 genes associated with antibiotic production and pellicle formation) also provided evidence that these regulatory systems play important roles in the formation of pellicle in *B. velezensis* CC09. For instance, the relative expression of *abrB* and *sinI*, which negatively regulate the expression of genes related to extracellular polysaccharide synthetases, dramatically increased in both the CC09-RIF3 (H485R) and CC09-RIF5 (S490L) mutants, leading to a defect in pellicle formation. The delayed formation and decreased biomass of pellicle in CC09-RIF5 (S490L) may have been caused by down regulation of the expression of *degS* and *degU*. This may in turn have resulted in a higher expression of *pgdS* (**Table 3**) and, consequently, a reduction in γ-PGA. It has reported that the production of γ-PGA is positively regulated by DegU/DegS and negatively regulated by PgdS (Ohsawa et al., 2009; Scoffone et al., 2013). Therefore, we speculate that the more severe defect in pellicle formation in CC09-RIF5 (S490L) compared to CC09-RIF3 (H485R) resulted from the down regulation of *degS* and *degU* together with the up regulation of *abrB* and *cheV.*

Compared to the WT strain, the Rif^r^ mutants exhibited various multicellular growth patterns (**Figure 2**). It has been reported that the formation of multicellular growth patterns is an exceptionally complex process (e.g., it involves cell–cell interaction, communication via chemotaxis signaling, and swarming motility) that requires the regulation of many genes at all levels of gene regulation (Martínez and Vadyvaloo, 2014). For example, the DegS/DegU regulatory system has been reported to control a variety of cell phenotypes, including extracellular protein production, swarming motility, pellicle formation, and the formation of complex structures based on multicellular growth patterns (Verhamme et al., 2007, 2009). The down regulation of *degS* and *degU* in the CC09-RIF5 (S490L) strain might have led, at least in part, to the morphological change in its multicellular growth pattern compared to the WT strain. However, the morphological change in the multicellular growth pattern of CC09-RIF3 (H485R) was not due to a change in *degS*/*degU* expression (**Table 3**), which indicates that bacterial multicellular growth patterns are regulated by more than one regulatory system. However, further studies are required to elucidate how the Rif^r^ mutations lead to changes in the multicellular growth patterns of *B. velezensis*.

## Author Contributions

X-CC contributes to the acquisition, analysis, interpretation of data and drafting the work, HX the isolation of spontaneous Rif^r^ mutants, LL the preparation of isogenic mutations, Y-RX the determination of specific growth rate, J-DL the observation of pellicle, C-HL the conception and design of the work and revision of the paper, and X-YY the conception of the work.

## Conflict of Interest Statement

The authors declare that the research was conducted in the absence of any commercial or financial relationships that could be construed as a potential conflict of interest.
