# Supplementary material for: Rifampicin-Resistance Mutations in the rpoB Gene in Bacillus velezensis CC09 have Pleiotropic Effects
Source: Front Microbiol. 2017 Feb 13;8:178. doi: 10.3389/fmicb.2017.00178 (PMC5303731; doi:10.3389/fmicb.2017.00178)
Supplement: Supplementary file 1 [file Presentation_1.pdf]

Table S1. Primers used for PCR amplification of the *rpoB* gene in *B. velezensis* CC09

Primers	Sequences
rpoB_r1f	5’-AGGTCAACTAGTTCAGTATGG-3’
rpoB_r1r	5’-TAATTCAGCAAGCGGGTTCG-3’
rpoB_r2f	5’-TCAGCTACTTCTTCAACCTC-3’
rpoB_r2r	5’-TTACCTACGAGAAGGTCTCC-3’
rpoB_r3f	5’-AGATCACCCGTGATATTCC-3’
rpoB_r3r	5’-TACTCTTTCGTTACTGCGTC-3’

Table S2. Primers used for PCR amplification of the sequence containing the point mutation in the *rpoB* gene of each mutant

Primer	Sequences
PMRif1	5’-TTAACTAGACAGATCTGATTCTCGATCTCATTGGTGAGAACG-3’
PMRif2	5’-GAAGCTTCTAGAATTCTACCTACGAGAAGGTCTCCGTCGTTG-3’
PMRif3	5’-CCTGTTTAGGA**A**ATACGTCCATG-3’
PMRif4	5’-TAT**T**TCCTAAACAGGTTGTATCTGCTGCGACAGCATG-3’

Underline indicates the sequences are homologous to the sequence of *rpoB* gene in *B. velezensis* CC09. The sequences without underline in PMRif1 and PMRif2 indicate these sequences are homologous to the left and right tails of the digested fragment of pMAD. The bold underlines in PMRif3 and PMRif4 indicate the point nucleotide mutation of S617F was prepared by PCR application using two pairs of primers PMRif1/PMRif3 and PMRif4 and PMRif2. The PCR product containing S617F was further ligated to pMAD and introduced into competent cells of *B. velezensis* CC09 to create a new point mutation.

Table S3. Pairs of primers targeting selected genes used for quantitative real-time PCR analysis

No.	Primer	Primer sequences	Gene	Protein	Related Function
**1**	degS-qf	GCATTTGTGACGGCTTCCTGAG	*degS*	Two-component sensor histidine kinase	Swarming motility, biofilm formation (Shemesh and Chai, 2013)
	degS-qr	AAGAAATTCGCAACGCCTATGA			
**2**	degU-qf	ATTGCCGGTTATCTGCTTCACG	*degU*	Two-component sensor histidine kinase	Swarming motility, biofilm formation (Shemesh and Chai, 2013)
	degU-qr	GTTCAATGCGGTTGTCTTCCTC			
**3**	ituA-qf	TGCGCCAGACCTTCAGTTTATG	*ituA*	Iturin A synthetase A	Antimicrobial cyclic lipopetides Itruin A (NCBI gene database)
	ituA-qr	GGGTATCTTGGCTATTTACAGCATCT			
**4**	ituB-qf	CCCGAATGACATCTACTAAGGTTTG	*ituB*	Iturin A synthetase B	Antimicrobial cyclic lipopetides Itruin A (NCBI gene database)
	ituB-qr	ATGCATCTGCCGTTCCTTATCT			
**5**	pgdS-qf	TGCTTGGATGAACCTTCCCTCT	*pgdS*	γ-DL-glutamyl hydrolase	Swarming motility, biofilm formation (Stanley and Lazazzera, 2005)
	pgdS-qr	AGCCGCCGTTTCACATTTACCT			
**6**	srfAA-qf	TGCTTGGATGAACCTTCCCTCT	*srfAA*	Surfactin synthetase SrfAA	Antimicrobial lipopetides surfactin (NCBI gene database)
	srfAA-qr	AGCCGCCGTTTCACATTTACCT			
**7**	srfAB-qf	GCAAGCATTGATTGAACACCAT	*srfAB*	Surfactin synthetase SrfAB	Antimicrobial lipopetides surfactin (NCBI gene database)
	srfAB-qr	GCTTTTCAGCATATCCTCGTCAG			
**8**	yczE-qf	GGTGCTGATGGGAATGTTTATTG	*yczE*	Transmembrane protein	Biosynthesis of bacillomycin D (Koumoutsi et al. 2007)
	yczE-qr	CTGCGGCAAGAATCGTCAGCTC			
**9**	abrB-qf	AAGTGCGATACCTTGCTGGTCA	*abrB*	Transition state regulatory protein	Biofilm formation (Hamon et al. 2004)
	abrB-qr	CACTGCTTATGCTCGGAGATGA			
**10**	sinI-qf	CAGAAAGGATTTACGGTATGAC	*sinI*	Transcriptional regulator	Biofilm formation(Verhamme et al. 2009)
	sinI-qr	TCGCAATTAGATAAGGAATGG			
**11**	sinR-qf	TAACTTCACGGACAAACACCACTG	*sinR*	Transcriptional regulator	Biofilm formation(Verhamme et al. 2009)
	sinR-qr	GAGCTTAAATAAGACTTCGCTACC			
**12**	cheV-qf	CAATCGCCTCCCAGGACACC	*cheV*	Response regulator for *CheA* activity	Chemotaxis, swarming motility (Kearns and Losick, 2003)
	cheV-qr	CGAAATTCTTCCCGTCATCAGT			

**13**	spo0A-qf	AGGTAAGCGGAAATGTCAGCAGT	*spo0A*	Response regulator	Biofilm and spore formation, etc. (Verhamme et al. 2009)
	spo0A-qr	TTTGGCGATGTCGGGATAAAGGA			
**14**	kinC-qf	GCAAGGCGTTCGTTCGTTTCCAG	*kinC*	Two-component sensor histidine kinase	Swarming motility, biofilm formation (Shemesh and Chai, 2013)
	kinC-qr	CTCCCAAATCAAGCAGCAGTCC			
**15**	kinE-qf	AGCTGACCATGAGACCGAGACC	*kinE*	Two-component sensor histidine kinase	Swarming motility, biofilm formation (Shemesh and Chai, 2013)
	kinE-qr	GCGGCAATATCTACGTGACCATTC			
**16**	16S-qf	TGTGGGATTGGCTTAACCTCG	*16S* rDNA	16S ribosomal RNA	16S ribosomal RNA
	16S-qr	TGTCGTCAGCTCGTGTCGTG			

Hamon, M. A., Stanley, N. R., Britton, R. A., Grossman, A. D., and Lazazzera, B. A. (2004). Identification of abrB-regulated genes involved in biofilm formation by Bacillus subtilis. Mol. Microbiol. 52, 847-860. doi: 10.1111/j.1365-2958.2004.
04023.x
Kearns, D. B., and Losick, R. (2003). Swarming motility in undomesticated Bacillus subtilis. Mol. Microbiol. 49, 581-590. doi: 10.1046/j.1365-2958.2003.03584.x
Koumoutsi, A., Chen, X. H., Vater, J., and Borris, R. (2007). DegU and YczE positively regulate the synthesis of bacillomycin D by Bacillus amyloliquefaciens strain FZB42. Appl. Environ. Microbiol. 73, 6953-6964. doi: 10.1128/AEM.00565-07
Shemesh, M., and Chai, Y. (2013). A combination of glycerol and manganese promotes biofilm formation in Bacillus subtilis via histidine kinase KinD signaling. J. Bacteriol. 195, 2747-2754. doi: 10.1128/JB.00028-13
Stanley, N. R., and Lazazzera, B. A. (2005). Defining the genetic differences between
wild and domestic strains of Bacillus subtilis that affect poly-g-DL-glutamic acid production and biofilm formation. Mol. Microbiol. 57, 1143-1158. doi: 10.1111/j.1365-2958.2005.04746.x
Verhamme, D. T., Murray, E. J., and Stanley-Wall, N. R. (2009). DegU and Spo0A jointly control transcription of two loci required for complex colony development by Bacillus subtilis. J. Bacteriol. 191, 100-108. doi: 10.1128/JB.01236-08

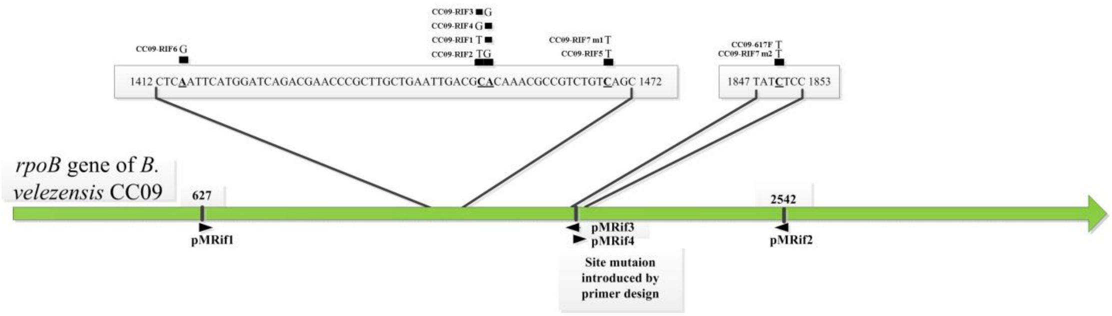

Fig. S1 Primer design covering each point mutation in the *rpoB* gene of *B. velezensis* CC09

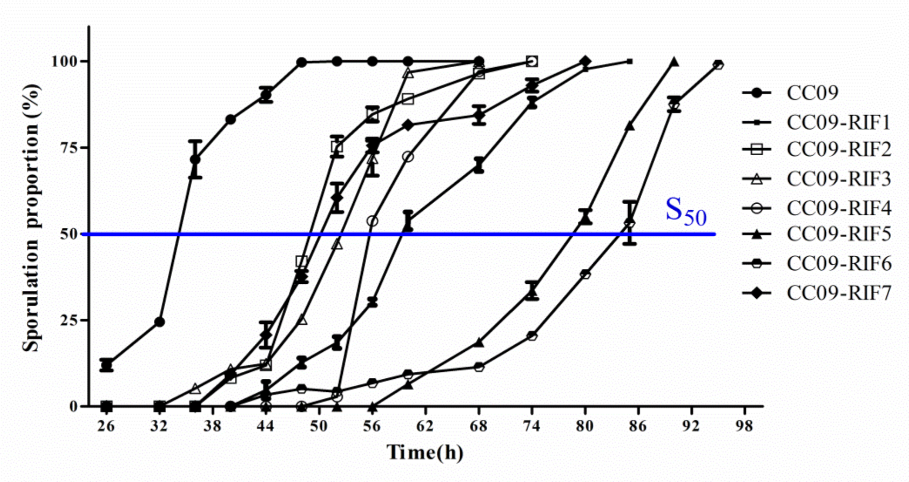

Fig. S2 Sporulation curves of the WT strain and Rif^r mutants

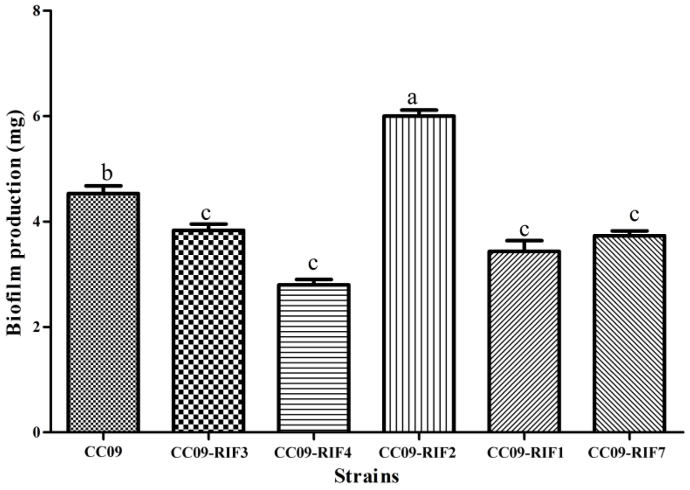

Fig. S3 Quantity of pellicle formed by the WT strain and Rif^r mutants

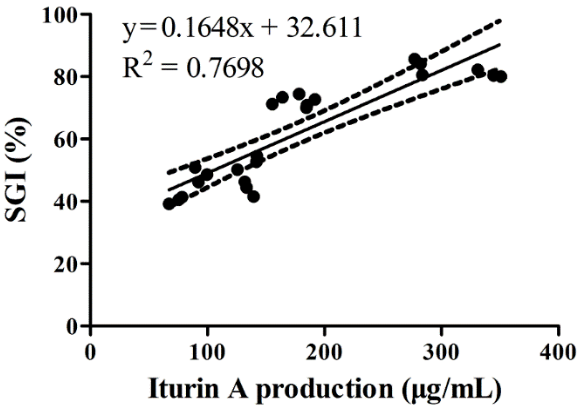

Fig. S4 Correlation between iturin A production and inhibition of fungal spore germination

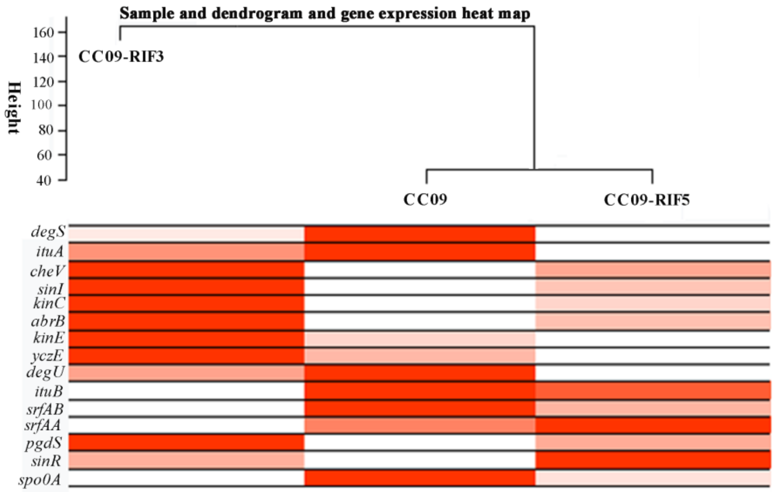

Fig. S5 Dendrogram of hierarchical clustering based on the relative expression of the selected genes
